# Pubertal development and screen time among South Korean adolescents: testing body mass index and psychological well-being as mediators

**DOI:** 10.1186/s41256-016-0019-2

**Published:** 2016-12-05

**Authors:** Eun-Young Lee, John C. Spence

**Affiliations:** 1 1-149 Van Vliet Complex, Faculty of Physical Education and Recreation, University of Alberta, Edmonton, AB T6G 2H9 Canada; 2 3-113 Van Vliet Complex, Faculty of Physical Education and Recreation, University of Alberta, Edmonton, AB T6G 2H9 Canada

**Keywords:** Puberty, Longitudinal study, Depression, Self-esteem, KCYPS

## Abstract

**Background:**

This study tested links between pubertal development and screen time among South Korean adolescent boys and girls.

**Methods:**

Secondary analysis was conducted on data from the Korean Children and Youth Panel Study (KCYPS) involving 2071 adolescents (*age M* = 13.14 years). Body mass index (BMI) at Grade 8 (baseline), self-esteem and depression at Grade 9 were examined as mediators of the relationship between pubertal development and screen time after adjusting for household income and academic performance. Structural equation modeling was used to assess direct and indirect pathways between pubertal development at Grade 8 and screen time at Grade 9.

**Results:**

No direct effect of pubertal development on screen time was found. But, an indirect effect existed for boys from pubertal development to screen time through BMI. Earlier pubertal development predicted higher BMI, and in turn, higher BMI predicted more time spent in screen time. Among girls, pubertal development negatively predicted BMI; however, no mediation effect of BMI between pubertal development and screen time was observed. No mediation effect of self-esteem or depression was found among boys or girls.

**Conclusions:**

Pubertal development appears to have an indirect influence on screen time through BMI for South Korean boys. More studies examining potential pathways between pubertal development and sedentary behavior are needed to build on these findings.

## Background

Accumulating evidence suggests that sedentary behavior (SB), as well as physical activity, is an important determinant of the current and future health of adolescents [1, 2]. Therefore, identifying predictors of SB should be a key public health priority. Screen time (i.e., time spent watching television, playing video/computer games, and surfing the internet while in a seated or reclined position) is the most common type of SB [1–3]. Notable individual-level correlates of screen time include age, sex, and weight status [1, 3]. Though biological factors such as maturation (e.g., pubertal development) are thought to influence physical activity among young people [4], only a few studies have examined the role of such factors on SB. For instance, more advanced puberty positively predicted screen time, which included time spent in watching television and playing video games, in a large sample of British adolescents aged between 12 and 13 years [5]. In addition, advanced pubertal development (i.e., percentage of predicted mature stature, which is the child’s current height relative to their expected height at maturity) was associated with higher accelerometry-measured SB among 13–16 year-old Portuguese boys [6]. Though evidence is limited, advanced pubertal development appears to be associated with high levels of screen or sitting time.

The mechanisms by which pubertal development influences SB are even less well understood. Because pubertal development is associated with weight status, particularly among girls [7, 8], and weight status is one of the correlates of SB [1], it is possible that pubertal development may influence screen time mediated by weight status. Likewise, puberty accompanies psychological changes [9], and psychological factors are known to be associated with SB among adolescents [10]. For instance, prolonged time spent in SB was associated with higher depressive symptoms [11], lower self-esteem [12], and less pro-social behavior [13] among children. Two longitudinal studies reported negative associations between screen time and self-esteem among boys, and a positive association between screen time and aggression among girls [14, 15]. Thus, psychological well-being may be another potential mediator of the relationship between pubertal development and SB.

Children with advanced pubertal development, among girls in particular, are at an increased risk for accelerated skeletal maturation, early sexual debut, potential sexual abuse, and psychosocial difficulties [9]. Furthermore, several epidemiological studies reported that early puberty is associated with an increased risk for breast cancer in girls and testicular cancer in boys, and at a higher risk for developing obesity, type 2 diabetes, and cardiovascular disease in adulthood [9]. In addition, mental disorders including depression, certain anxiety disorders, eating disorders, and substance use disorders has been reported to associated with early pubertal development, rather than chronological age per se [9]. Therefore, it is suggested that health and social services professionals working with adolescents and schools should focus on those who mature earlier than their counterparts. Given the growing concern associated with puberty and related health problems that may track into adulthood [9], preventive strategies should focus on modifiable behaviors relevant to health. There is an established body of research of potential mediators on the associations between pubertal development and physical activity [4]. Our study fills a gap in the literature by focusing on screen time, another highly relevant but less studied health behavior for emerging adolescents.

The aim of this study, therefore, was to examine whether BMI and psychological well-being mediate an association between pubertal development and screen time among South Korean adolescents. Because patterns of screen time may differ between the sexes [16], data for boys and girls were analyzed separately. Specific hypotheses included: (i) Early pubertal development will cross-sectionally predict higher BMI at Grade 8 and, in turn, longitudinally predict higher screen time at Grade 9; (ii) early pubertal development at Grade 8 will cross-sectionally predict higher BMI at Grade 8 and, in turn, longitudinally predict lower psychological well-being, and higher screen time at Grade 9; and (ii) early pubertal development at Grade 8 will longitudinally predict higher screen time at Grade 9 mediated by higher BMI at Grade 8 and lower psychological well-being at Grade 9. Understanding the potential mechanisms linking puberty and screen time may help researchers to develop targeted interventions (e.g., by pubertal development stage) aimed at reducing screen time among South Korean adolescents.

## Methods

### Participants

This study involved a secondary analysis of data from the KCYPS, which is a seven-year prospective panel study of a representative sample of students in Grade 1, 4, and 7 in South Korea [17]. Each panel aimed to recruit at least 2200 students using a stratified multi-stage cluster sampling method in 2009; the sample population was geographically representative of Korea. KCYPS was developed based on Bronfenbrenner’s ecological framework [18]; the survey questions are divided into two categories (i.e., personal development and environment) with 10 sub-categories (physical, intellectual, socio-emotional development, delinquency behavior, and lifestyle patterns). Data were available and accessible to any registered member of the National Youth Policy Institute (NYPI) data archives website (http://archive.nypi.re.kr/). For this study, waves 2 and 3 of the Grade 7 panel were selected because wave 2 was the only one that included the assessment of pubertal development and wave 3 had all outcome variables.

At baseline, four trained research staff visited participating schools during regular school hours. Self-report questionnaires were administered to the students who were encouraged to complete all items. Demographic information was collected from the caregivers of each student. For the Grade 7 panel, baseline measures were collected from October to November in 2009 (wave 1). Subsequent measures were made from October to December in every year since 2010 (wave 2). Students who participated in the survey at baseline, and who agreed to continue in the study were contacted via telephone at follow-up. After obtaining a verbal consent, research staff then had a face-to-face meeting with each student followed by an interview with caregivers to collect demographic information. According to the NYPI data user manual, incentives were provided to students who participated in each panel [17].

At the first wave in 2010, data were collected from a total of 2351 students (49% of females). Follow-up rates for the second and third waves were 97.0% (*N* = 2346) and 96.1% (*N* = 2337) respectively. For the current study, the second and third waves (2011–2012) were downloaded and analyzed because they were the only ones to include measures of pubertal development and psychological well-being along with the other necessary variables. After excluding cases with missing data, the total number of eligible students included in the analyses were 2071 (49% of females). No significant differences were found in the testing variables before and after excluding missing cases (*p* > .05). The details of the goals, design, and sampling of this panel were published on the data archives webpage of NYPI (http://archive.nypi.re.kr/) [19]. Ethics approval was obtained by Statistics Korea for baseline data collection and for the follow-up data collection (approval number: 40202). Prior to data collection, informed written consent was obtained from the parent/main guardian of each student and verbal assent was obtained from the participants. Furthermore, all data in the KCYPS is de-identified [17].

### Measures

#### Pubertal development

At wave 2 (Grade 8), participants were asked in which grade they first experienced ejaculation (i.e., semenarche, spermarche, oiarche)/menstruation. Responses options were as follows: 1 (have not yet experienced), 2 (Grades 1–3), 3 (Grade 4), 4 (Grade 5), 5 (Grade 6), 6 (Grade 7), and 7 (Grade 8). For analyses, the scores were recoded so that the lowest score represented late pubertal development and the highest score represented early pubertal development (i.e., 0 = no menarche/semenarche; 1 = Grade 8; 2 = Grade 7; 3 = Grade 6; 4 = Grade 5; 5 = Grade 4; 6 = Grade 1–3). Menarche and semenarche have been recognized as landmark pubertal events in each sex; they are a key part of childhood and adolescence in all biological, psychological, social, and cultural realms [20].

#### Body mass index (BMI)

At waves 2 (Grade 8) and 3 (Grade 9), weight and height were self-reported by participants. BMI was computed by dividing weight in kilograms by height in meters squared (kg/m^2^).

#### Psychological well-being indicators

Korean versions of the Beck Depression Inventory [21] and the Rosenberg Self-esteem Scale [22], translated and validated by the Korea University Behavioral Science Research Institute [23], were used to assess depression at waves 2 (Grade 8) and 3 (Grade 9), and self-esteem at wave 3 (Grade 9) among participants. The scales consisted of 10 items each and the responses were scaled from 1 (very much) to 4 (not at all). Depression and self-esteem scores were recoded so that lower scores indicated negative psychological well-being. In the current sample, the internal consistency coefficients for depression at Grades 8 and 9, were α = .90, and .91 respectively. The corresponding Cronbach’s alpha for self-esteem at Grade 9 was .60. Thus, exploratory factor analysis was performed to improve the internal consistency of the self-esteem measure and determine the most appropriate item structure for the sample.

#### Screen time

Screen time was measured by four questions asking students to report the amount of time spent (hour and minute) in playing computer/video games, and watching television during weekdays (two items), and on weekends (two items) at waves 2 (Grade 8) and 3 (Grade 9). For the analysis, screen time in each category was calculated by converting hours to minutes and then adding all screen time minutes during weekdays and weekend days.

#### Covariates

Monthly household income reported by the parents/guardians of all participating students at wave 3 (Grade 9) was categorized into quartiles (in Korean Won [KRW]; 1000 KRW is the equivalent of USD 0.88): Quartile 1 = lowest-2,999,999; quartile 2 = 3,000,000–4,199,999; quartile 3 = 4,200,000-5,999,999; and quartile 4 = 6,000,000-highest. Satisfaction with academic performance was obtained by asking participants to rate their achievement at school at wave 2 (Grade 8) with response options scaled from 1 (very satisfied) to 5 (not satisfied at all).

### Statistical analysis

All data were screened and checked for inconsistencies by KYCPS before being released. An exploratory factor analysis was performed on the self-esteem measures. The principal components method with an oblique rotation was used to allow for a correlated factor structure. A number of factor extractions were attempted to achieve an acceptable internal consistency (α = .80); 4 out of 10 items were removed from the self-esteem measure. Because the pubertal development scores were not normally distributed, they were log transformed for the analyses.

To examine the patterns of BMI, psychological well-being, and screen time (hypothesis one), descriptive statistics were tabulated; sex differences within the same grade, and grade-differences within the same sex were also calculated through paired sample t-tests. To test hypotheses two and three, bivariate associations between pubertal development in Grade 8, BMI, psychological well-being, and screen time in Grades 8 and 9, self-esteem in Grade 9, household income, and academic performance were assessed using Spearman rank correlation analysis (*ρ*). The strength of a correlation was determined based on the following criteria: *ρ* = .00–.19 *very weak*; *ρ* = .20–.39 *weak*; *ρ* = .40–.59 *moderate*; *ρ* = .50–.79 *strong;* and *ρ* = .80–1.00 *very strong* [24]*.* The 0.95 confidence intervals (CI) for ρ were calculated based on the Fisher r-to-z transformation.

Consequently, structural equation modeling (SEM) was conducted using maximum likelihood to fit the model. Because the data violated a multivariate normality assumption, the SEM analysis was conducted using bootstrapping procedures with 5000 resampling separately for depression and self-esteem with IBM SPSS AMOS software. Separate analysis was conducted to test the model by gender. The model potentially explaining the relationship between pubertal development and screen time included the following pathways: (i) an association between pubertal development and BMI at wave 2 (Grade 8); (ii) an association between BMI at wave 2 (Grade 8) and screen time at wave 3 (Grade 9); (iii) an association between BMI at wave 2 (Grade 8) and psychological well-being at wave 3 (Grade 9) after adjusting for corresponding psychological well-being at wave 2 (Grade 8); (iv) an association between psychological well-being and screen time at wave 3 (Grade 9) after adjusting for screen time at wave 2 (Grade 8). All analyses were also adjusted for parental-reported household income and self-reported academic performance at wave 3 (Grade 9). The SEM approach uses latent variables that represent a hypothetical construct with multiple measurable variables. It allows for inclusion of multiple variables of the same construct without problems of collinearity and accounts for measurement error of each indicator [25]. The root mean square error of approximation (RMSEA) and comparative fit index (CFI) were used to determine the approximate fit of the model, the improvement in fit over the null model and the fit adjusted for parsimony with independence of sample size, respectively [26]. RMSEA values close to .05 (or lower), and CFI values of close to .95 reflect good fit between the model and data [26]. In accordance with recent recommendations [27], mediated effects were explored by examining the 90% upper and lower limits of bootstrap-generated bias-corrected confidence intervals (BBC 90% CI) of indirect effects.

## Results

Table 1 shows various characteristics of the study sample including physical attributes and screen time. Boys were generally taller and heavier than girls both at Grade 8 and 9. As for pubertal development, 21.6% of boys and 6.1% of girls had not experienced semenarche/menarche at baseline. Depressed mood increased with age, and girls scored higher than boys at Grade 8 (3.15 ± 0.61 in boys vs. 3.11 ± 0.61 in girls) and 9 (2.99 ± 0.61 in boys vs. 2.93 ± 0.63 in girls). Self-esteem was higher among boys (2.84 ± 0.45) than girls (2.80 ± 0.01). Time spent in screen time decreased over time for both boys and girls. Boys spent more time in screen time than girls at Grades 8 and 9 (123.05 ± 55.77 min/day and 114.62 ± 55.21 min/day in boys vs. 121.66 ± 54.48 min/day and 109.38 ± 60.79 min/day in girls).

**Table 1:** Descriptive statistics for physical characteristics, pubertal development, screen time, and depression at Grades 8 and 9— Korea Children and Youth Panel Study, 2011–2012

	Scale	Grade 8 (*n* = 2276)		Grade 9 (*n* = 2226)	
		Boys	Girls	Boys	Girls
N (%)		1150 (50.5)	1126 (49.5)	1128 (50.7)	1098 (49.3)
Household income (1000 Korean won = USD 0.88) (%)					
Q1 (lowest-2,999,999)		–	–	21.0	20.4
Q2 (3,000,000–4,199,999)		–	–	30.9	27.8
Q3 (4,200,000–5,999,999)		–	–	21.3	25.5
Q4 (6,000,000–highest)		–	–	26.8	26.3
Academic performance (M ± SD)	1–5	–	–	2.75 ± 0.82	2.76 ± 0.78
Height (M ± SD)		167.58 ± 7.09	159.41 ± 5.35^*^	**171.26** ± **6.31**	**160.42** ± **5.30** ^*^
Weight (M ± SD)		57.88 ± 11.62	50.18 ± 7.70^*^	**61.25** ± **11.24**	**51.59** ± **7.15** ^*^
BMI (M ± SD)		20.53 ± 3.49	19.73 ± 2.74^*^	**20.85** ± **3.39**	**20.02** ± **2.51** ^*^
Age (M ± SD)		12.15 ± 0.60	12.10 ± 0.59	13.16 ± 0.59	13.11 ± 0.63
Pubertal development (M ± SD)	0–6	1.27 ± 1.32	2.63 ± 1.12*	–	–
Depression (M ± SD)	1–5	3.15 ± 0.61	3.11 ± 0.61	**2.99 ± 0.61**	**2.93 ± 0.63**
Self-esteem (M ± SD)	1–5	–	–	2.84 ± 0.45	2.80 ± 0.01^*^
Screen time during weekdays (min)^a^ (M ± SD)					
Computer/video games		86.16 ± 65.88	84.35 ± 66.63	88.57 ± 67.11	**69.83 ± 67.29**
TV watching		89.59 ± 66.87	96.78 ± 73.13^*^	**84.06 ± 63.43**	94.73 ± 75.38^*^
Screen time on weekends (min)^a^ (M ± SD)					
Computer/video games		162.48 ± 104.59	135.15 ± 93.91	**157.88 ± 102.58**	**114.87 ± 93.35** ^*^
TV watching		153.97 ± 95.95	170.37 ± 104.97	**137.06 ± 89.12**	168.91 ± 107.17^*^
Screen time total (min)^a^ (M ± SD)		123.05 ± 55.77	121.66 ± 54.48	**114.62 ± 55.21**	**109.38 ± 60.79** ^*^

Data were presented as means and standard deviations (M ± SD) or percentages (%)

^a^Screen time: Computer/video games, watching television

^*^ *p* < 0.05, significant sex differences within the same grade

***p*** **< 0.05**, significant differences over time within sex

Bivariate correlations between predictor and outcome variables and covariates are shown in Table 2 separately by sex. Among boys, pubertal development at Grade 8 showed very weak correlations with BMI in Grade 8 (*ρ* = .10; 95% CI = .04, .16) and 9 (*ρ* = .11; 95% CI = .05, .17), pubertal development was not correlated with either well-being indicators or screen time. Among girls, pubertal development at Grade 8 was correlated with BMI at Grade 8 (*ρ* = .33; 95% CI = .28, .38) and 9 (*ρ* = .32; 95% CI = .26, .37). In both sexes, psychological well-being indicators at Grades 8 and 9 were weakly to moderately correlated with each other (*ρ* = .38 ~ .43 among boys and *ρ* = .45 ~ .54 among girls). Marginally significant correlations were observed between screen time, and psychological well-being indicators.

**Table 2:**
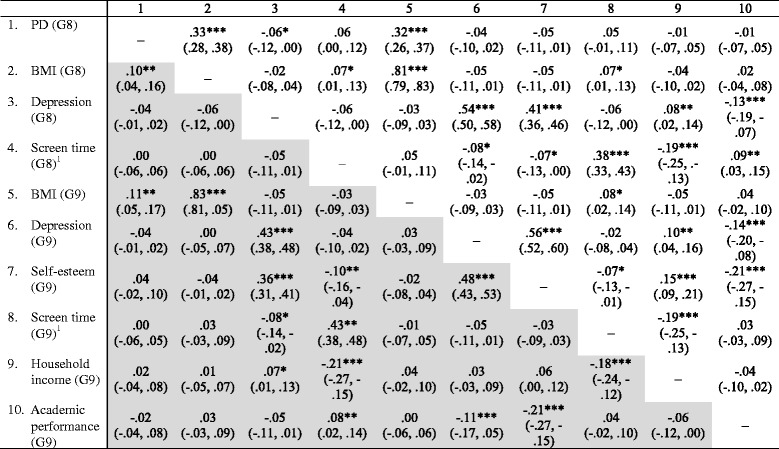
Correlation coefficients (95% confidence intervals) for pubertal development (PD), Body Mass Index (BMI), psychological well-being, and screen time among boys (*n* = 1056) (shaded) and girls (*n* = 1015)—Korea Children and Youth Panel Study, 2011–2012 (*N* = 2071)

^1^Screen time: Computer/video games, television viewing

***p < 0.001, **p < 0.01, *p < 0.05

**Fig. 1:**
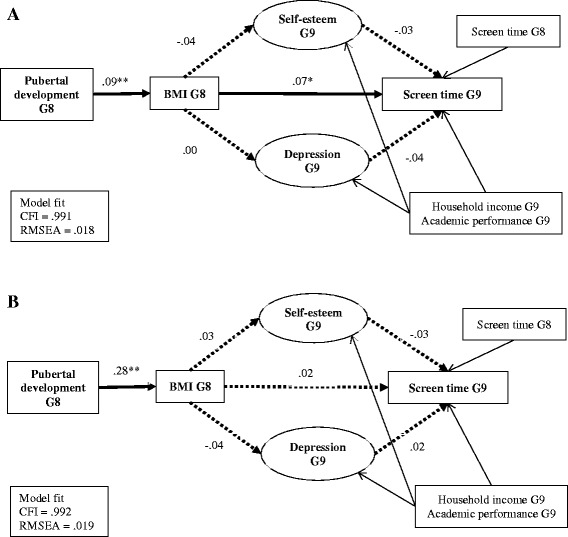
Associations between pubertal development, Body Mass Index (BMI), psychological well-being and screen time—Korea Children and Youth Panel Study, 2011–2012 (*N* = 2071). **a**. Mediated effects (β) model describing the associations between pubertal development, Body Mass Index (BMI), psychological well-being and screen time among boys (*n* = 1056). **b**. Mediated effects (β) model describing the associations between pubertal development, Body Mass Index (BMI), psychological well-being and screen time among girls (*n* = 1015). The primary variables of interest are shown in bolded texts; covariates are shown in non-bolded texts. Bolded lines indicate significant effect; dashed lines indicate non-significant effect; non-bolded lines indicate covariates. A rectangle represents a measured variable and an oval represents a latent variable. CFI = Comparative Fit Index; RMSEA = Root Mean Squared Error of Approximation. ***p < 0.001, **p < 0.01

The results of mediation analyses are shown in Fig. 1, separately by sex. Latent variables were created for depression and self-esteem measures to limit the number of estimated parameters using item parceling [28]. One of the advantages of parceling items is that the composite-level indicators tend to be more reliable and normally distributed, and to have values that are more continuously distributed. In addition, the number of indicators in mediation models directly affects the sample size requirements [28].

The 10 items of the depression measure were parceled into five indicators of a latent variable representing depression. Item-to-construct loadings were used to pair items (e.g., the highest and the lowest loadings, the second highest and the second lowest loadings…the fifth and sixth loadings). The same strategy was employed for the self-esteem measures, producing three indicators. Figure 1 shows the model fit and standardized Beta coefficients associated with the model separately by sex. The mediated models for boys and girls showed adequate fit between the proposed model and the data (RMSEA = .018, CFI = .991 among boys; RMSEA = .019, CFI = .992 among girls).

For the models assessing the pathways between pubertal development and screen time, an indirect relationship existed between pubertal development and screen time through BMI among boys. Specifically, advanced puberty among boys compared to their peers was associated with higher BMI (β = .09; BBC 90% CI = .04, .14), and in turn, predicted a greater amount of time spent in screen time (β = .07, BBC 90% CI = .02, .12), independent of the specified covariates. Among girls, pubertal development positively predicted BMI (β = .28, BBC 90% CI = .23, .34) but no mediation effect between pubertal development and screen time was observed.

## Discussion

This study examined mediation models depicting the associations between pubertal development, BMI, psychological well-being (i.e., depression, self-esteem), and screen time separately by sex among South Korean adolescents. Though the amount of screen time decreased over time among both boys and girls, sex differences existed. In addition, we found sex differences in the relationship between pubertal development and screen time. Notably, boys spent significantly more time in screen time than girls at Grade 9, but not at Grade 8. Among boys, an indirect influence of pubertal development on screen time through BMI was observed. Neither depression nor self-esteem mediated the association between pubertal development and screen time.

The key finding of this study is the role that BMI appears to play for boys. Though no direct effect of pubertal development on screen time was found, pubertal development positively predicted BMI at Grade 8, and BMI positively predicted screen time (e.g., watching television, playing computer or video games) at Grade 9 after adjusting for other specified covariates. According to Hayes [29], it is possible for a mediator (M) to be causally linked between a predictor (X) and a criterion (Y) variable even when no association exists between X and Y; in such cases, it is suggested to use the term *indirect effect* instead of *mediator* to describe the relationship [30]. These results suggest that higher BMI may be also a marker for, not just a result of [14, 31], extended screen time. For instance, a critical aspect of puberty is the maturation of the hypothalamic-pituitary-gonadal axis which mediates the release of gonadotrophins and ultimately results in changes in body size and composition. This may in turn directly or indirectly influence human behaviors associated with energy expenditure because the hypothalamus is a key structure in regulating physical activity [32]. It is also important to note that high BMI among boys can be misleading. Specifically, an increased muscle mass, rather than an increase in adiposity in male puberty may increase BMI [8].

No direct relationship was observed between pubertal development and screen time in our study. Similarly, Bradley and colleagues [33] reported that pubertal status was not a significant predictor of screen time among middle school boys. In contrast, a longitudinal examination of maturation and video game playing found that late and post-pubertal boys were more sedentary than boys in mid-puberty (mean age = 15 years) [34]. Also, pubertal maturation was a significant predictor of SB among Portuguese boys [6] and screen time among British boys [5]. Future research should test for both direct and indirect effects of pubertal development on screen time or screen-based SB. Furthermore, it is possible that cultural factors may moderate the role or impact of biological processes (e.g., pubertal development) on engagement in an excessive consumption of screen time [4]. For instance, in comparison to Chinese and Japanese adolescents, more South Korean adolescents identify computer-based screen time as their most preferred leisure time activity [35]. Furthermore, internet addiction is a rising issue among South Korean teenagers [36]. Future studies are required to gain further understanding about a potential moderating effect of cultural factors.

One of the possible explanations as to why neither depression nor self-esteem mediated the relationship between pubertal development and screen time in our study may be due to *suppression* [25]. Specifically, the beta coefficients between pubertal development and BMI, between BMI and self-esteem, and between self-esteem and screen time among girls were positive, negative, and positive thus, opposing mediational processes may exist. It has been reported that such inconsistent mediation is more common in models with multiple mediator [25]. Another surprising finding that applied to both boys and girls in our study was that screen time decreased between Grades 8 and 9. Previous research suggests that, regardless of sex, older adolescents spend more time in SB than younger adolescents [3, 5, 37]. A longitudinal investigation also confirmed age-related increases in screen time across adolescence [31]. Though recreational screen time decreased over a one-year period among our sample, it is likely that South Korean adolescents are simply replacing their recreational SB with school-related SB. South Korea is well known for its “education fever”, in which secondary school students spend most waking hours in studying. For instance, despite a recent Korean government-imposed curfew of 10:00 pm on educational institutes, students, particularly those in higher grades, spend approximately 15 hours daily (often 8:00 am to 11:00 pm) in studying [38].

Puberty is a phase where adolescents are susceptible to an increased risk for many health and behavioral issues [9]. Public health strategies to promote adolescent health have predominantly focused on preventing adolescents from engaging in risky behaviors including the early initiation of sexual activities, smoking, alcohol consumption, and substance use. Similarly, limiting SB can also be an important health promoting strategy given the well-established effects on the health of children and adolescents [1, 2]. Though our study demonstrated the potential influence of early pubertal development on high screen time among boys, evidence is lacking. This is particularly important in that screen time is one of the most common risk behaviors among adolescents in modern societies.

A key strength of this study was the use of two-year follow-up data with a geographically representative, large sample of South Korean adolescents. In addition, this is the first study examining the association between pubertal development and screen time longitudinally among adolescents with potential mediators. Furthermore, the findings make important contributions to our understanding of screen time consumption among South Korean adolescents who are understudied in the literature regarding puberty and behavior. Regardless, some limitations should be acknowledged as well. The data were collected using self-reports from students and their parents, and inaccurate recall and intra personal bias may have occurred. In particular, self-reports of puberty related events are likely sensitive topics for adolescents to report. The only available indicator of pubertal maturation in the KCYPS was menarche in girls and semenarche in boys. Moreover, the use of objective or direct measures of pubertal development will help to elucidate this association in future investigation. The results might have been different if other maturation markers such as breast or pubic hair development were objectively measured. However, it is often difficult to obtain consent to objectively measure such markers and it would not be feasible in a large panel study of over 2000 students. Finally, self-esteem was measured at Grade 9 only; thus, we were not able to control for self-esteem at Grade 8.

## Conclusions

In summary, pubertal development appears to have an indirect influence on screen time through BMI for Korean boys. Public health efforts to reduce screen time among South Korean boys should target those with early pubertal development and higher BMI. This study contributes to the current literature, as only a few have examined such relationships among boys in particular. More studies examining potential pathways between pubertal development and screen time are needed to build on these findings.

## Abbreviations

BBC 95% CI: Bootstrap-generated bias corrected 95% confidence interval
BMI: Body mass index
CFI: Comparative fit index
KCYPS: Korea children and youth panel study
NYPI: National youth policy institute
RMSEA: Root mean square error of approximation
SB: Sedentary behavior

## References

[1] Salmon J, Tremblay MS, Marshall SJ, Hume C (2011). Health risks, correlates, and interventions to reduce sedentary behavior in young people. Am J Prev Med.

[2] Spence JC, Dinh Thy. Moving ahead: Taking steps to reduce physical inactivity and sedentary behaviour. Ottawa: The Conference Board of Canada, 2015. http://www.conferenceboard.ca/e-library/abstract.aspx?did=7022 Accessed 15 May 2015.

[3] Pate RR, Mitchell JA, Byun W, Dowda M (2011). Sedentary behaviour in youth. Brit J Sports Med.

[4] Cumming SP, Sherar LB, Pindus DM, Coelho-e-Silva MJ, Malina RM, Jardine PR (2012). A biocultural model of maturity-associated variance in adolescent physical activity. Int Rev Sport Exerc Psychol.

[5] Brodersen NH, Steptoe A, Boniface DR, Wardle J (2007). Trends in physical activity and sedentary behaviour in adolescence: ethnic and socioeconomic differences. Brit J Sports Med.

[6] Rodrigues AMM, Coelho-e-Silva MJ, Mota J, Cumming SP, Sherar LB, Neville H (2010). Confounding effect of biologic maturation on sex differences in physical activity and sedentary behavior in adolescents. Pediatr Exerc Sci.

[7] Davison KK, Susman EJ, Birch LL (2003). Percent body fat at age 5 predicts earlier pubertal development among girls at age 9. Pediatrics.

[8] Kaplowitz PB (2008). Link between body fat and the timing of puberty. Pediatrics.

[9] Patton GC, Viner R (2007). Pubertal transitions in health. Lancet.

[10] Tremblay MS, LeBlanc AG, Kho ME, Saunders TJ, Larouche R, Colley RC (2011). Systematic review of sedentary behaviour and health indicators in school-aged children and youth. Int J Behav Nutr Phys Act.

[11] Maras D, Flament MF, Murray M, Buchholz A, Henderson KA, Obeid N (2015). Screen time is associated with depression and anxiety in Canadian youth. Prev Med.

[12] Russ SA, Larson K, Franke TM, Halfon N (2009). Associations between media use and health in US children. Acad Pediatr.

[13] Mistry KB, Minkovitz CS, Strobino DM, Borzekowski DL (2007). Children’s television exposure and behavioral and social outcomes at 5.5 years: does timing of exposure matter?. Pediatrics.

[14] Epstein LH, Roemmich JN, Robinson JL, Paluch RA, Winiewicz DD, Fuerch JH (2008). A randomized trial of the effects of reducing television viewing and computer use on body mass index in young children. Arch Pediatr Adolesc Med.

[15] Neumark-Sztainer D, Goeden C, Story M, Wall M (2004). Associations between body satisfaction and physical activity in adolescents: Implications for programs aimed at preventing a broad spectrum of weight-related disorders. Eat Disord.

[16] Byun W, Dowda M, Pate RR (2012). Associations between screen-based sedentary behavior and cardiovascular disease risk factors in Korean youth. J Korean Med Sci.

[17] National Youth Policy Institute. Korea Children and Youth Panel Study (KCYPS) User’s Guide. 2014. http://archive.nypi.re.kr/. Accessed 20 Nov 2014.

[18] Bronfenbrenner U (1979). Contexts of child rearing: problems and prospects. Am Psychol.

[19] Kim JK, Baek HJ, Im HJ, Lee KO (2010). Korea children and youth panel study 2010-I.

[20] Ponton LE, Judice S (2004). Typical adolescent sexual development. Child Adolesc Psychiatr Clin N Am.

[21] Beck AT, Steer RA, Carbin MG (1988). Psychometric properties of the beck depression inventory: twenty-five years of evaluation. Clin Psychol Rev.

[22] Rosenberg M (1979). Conceiving the Self.

[23] Korea University Behavioral Science Research Institute (2000). Handbook of scales in psychology.

[24] Evans JD (1996). Straightforward statistics for the behavioral sciences.

[25] MacKinnon DP, Fairchild AJ, Fritz MS (2007). Mediation analysis. Ann Rev Psychol.

[26] Keith TZ (2014). Multiple regression and beyond: an introduction to multiple regression and structural equation modeling.

[27] MacKinnon DP, Lockwood CM, Williams J (2004). Confidence limits for the indirect effect: distribution of the product and resampling methods. Multivar Behav Res.

[28] Hall RJ, Snell AF, Foust MS (1999). Item parceling strategies in SEM: investigating the subtle effects of unmodeled secondary constructs. Organ Res Methods.

[29] Hayes AF (2009). Beyond baron and Kenny: statistical mediation analysis in the new millennium. Commun Monogr.

[30] Mathieu JE, Taylor SR (2006). Clarifying conditions and decision points for mediational type inferences in organizational behavior. J Organ Behav.

[31] Mitchell JA, Pate RR, Beets MW, Nader PR (2013). Time spent in sedentary behavior and changes in childhood BMI: a longitudinal study from ages 9 to 15 years. Int J Obesity.

[32] Eisenmann JC, Wickel EE (2009). The biological basis of physical activity in children: revisited. Pediatr Exerc Sci.

[33] Bradley CB, McMurray RG, Harrell JS (2000). Deng S changes in common activities of 3rd through 10th graders: the CHIC study. Med Sci Sports Exerc.

[34] Janz KF, Mahoney LT (1997). Maturation, gender, and video game playing are related to physical activity intensity in adolescents: the Muscatine study. Pediatr Exerc Sci.

[35] Lee EY, Yi KJ, Walker GJ, Spence JC (2016). Preferred leisure type, value orientations, and psychological well-being among East Asian youth. Leisure Sci.

[36] Kim JE, Kim J (2015). International note: teen users’ problematic online behavior: using panel data from South Korea. J Adolesc.

[37] Kim B (2010). Factors associated with physical activity and sedentary behavior among elementary school students. Korean J Health Educ Promot.

[38] ICEF Monitor. High performance, high pressure in South Korea’s education system. http://monitor.icef.com/2014/01/high-performance-high-pressure-in-south-koreas-education-system/. Accessed 23 Jan 3014.

